# Visual working memory as the substrate for mental rotation: A replication

**DOI:** 10.3758/s13423-024-02602-4

**Published:** 2024-12-02

**Authors:** W. Miro Ebert, Leonardo Jost, Petra Jansen, Biljana Stevanovski, Daniel Voyer

**Affiliations:** 1 Institute of Sports Sciences, University of Regensburg, Universitätsstraße 31, 93053 Bavaria, Germany; 2 University of New Brunswick, Fredericton, Canada

**Keywords:** Mental rotation, Visual working memory, Human sex differences

## Abstract

An experimental study by Hyun and Luck suggests that object working memory, but not spatial working memory, is employed during mental rotation. In contrast, correlational research points to the relevance of spatial working memory in mental rotation. Considering these somewhat conflicting results and the fact that a small sample was acquired in the study of Hyun and Luck, a replication of their study was conducted. Additionally, potential sex effects were explored. We collected (usable) data from 213 individuals across two experiments. All participants performed a mental-rotation task alone, a working-memory task alone, and both tasks concurrently. We expected greater rotation-dependent interference between tasks when the working memory task concerned object features (Experiment 1) than when it concerned spatial locations (Experiment 2). In Experiment 1, dual-task interference was observed in working-memory accuracy. In Experiment 2, there were interference effects in both mental rotation accuracy and working-memory accuracy. However, interference did not differ between experiments. Moreover, interference was not rotation dependent in either of the experiments. Thus, we could not replicate the findings of Hyun and Luck. No sex differences were found in exploratory analyses. The general interference effects found in this study may reflect the involvement of visual working memory in the processing and decision-making stages of the mental rotation of letters. This study underscores the need for further research to fully understand the role of visual working memory in mental rotation, especially with more complex stimuli.

**Supplementary Information:**

The online version contains supplementary material available at 10.3758/s13423-024-02602-4.

Mental rotation is thought to entail several stages of processing: the perceptual stages (perceptual processing, identification and discrimination of stimuli, identification of orientation), stages of the rotation process itself (mental rotation, judgment of parity), and decision-processing stages (response selection, execution; Heil & Rolke, 2002; Just & Carpenter, 1985; Shepard & Cooper, 1982). Especially in the later perceptual stages, visual working memory should play a crucial role because the object must be kept in mind and manipulated. Several studies provide empirical evidence to support this notion. For instance, electrophysiological evidence suggests the involvement of visual short-term memory in mental rotation (Prime & Jolicœur, 2010). Focusing on an event-related potential component connected with the maintenance of information in visual short-term memory, Prime and Jolicœur (2010) found increased offset latencies with increased angular disparity during mental rotation, indicating more extended maintenance of targets that are more strongly rotated.^1^

There is also behavioral evidence regarding the role of visual working memory in mental rotation. Visual working memory is believed to have two subcomponents: object working memory (concerned with object features) and spatial working memory (concerned with object location). This idea is supported by neuroscientific and behavioral data (e.g., Wood, 2011). To investigate how these subcomponents may be involved in mental rotation, Hyun and Luck (2007) used a dual-task paradigm. Specifically, they conducted two experiments where participants performed mental rotation of letters presented in canonical or mirrored form while completing a secondary visual working memory task. In their first experiment, Hyun and Luck used a secondary task concerned with object color. In the second experiment, the secondary task targeted spatial working memory. Both experiments used a concurrent articulatory suppression task to deter participants from verbally encoding to-be-remembered visual information (Besner et al., 1981). Hyun and Luck reported a significant interference effect (i.e., larger secondary task cost) in Experiment 1 (object working memory) but not Experiment 2 (spatial working memory) on both response times and accuracy in the mental-rotation task, with accuracy showing a rotation-dependent interference effect. Accuracy in secondary task performance showed a significant interference effect in both experiments, although the effect was rotation-dependent only in Experiment 1 (object working memory). Taken together, the studies of Prime and Jolicœur (2010) and Hyun and Luck highlight the role of visual working memory during mental rotation. However, the latter warrants the more specific claim that a representation of an object itself is maintained in memory rather than its spatial attributes.

The findings reported by Hyun and Luck (2007) have often been cited to support the particular importance of the visual information (i.e., representation of object) preserved in working memory during mental rotation (e.g., Göksun et al., 2013; Hollingworth & Rasmussen, 2010). A question in this context is whether spatial representations (stored in spatial working memory) are also relevant during the rotation process. Though Hyun and Luck acknowledge a spatial component to mental rotation, they claim that this component would be independent of the mechanism required to store object information during rotation.

Although, experimentally, no interference of a concurrent spatial working memory task with mental rotation performance was found, correlational data suggest the relevance of this system in mental rotation and other spatial abilities (e.g., Kaufman, 2007; Lehmann et al., 2014). Kaufman (2007) observed strong correlations between performance on spatial working memory tasks and a mental rotation test (Vandenberg & Kuse, 1978). Moreover, rotation block and verification block span entirely mediated sex differences in spatial test performance, although a unique contribution of sex to mental rotation performance remained. The author proposed that “spatial working memory capacity is the driving force determining the sex difference in mental rotation and spatial visualization ability” (Kaufman, 2007, p. 290). However, the approach used by Kaufman confounded visual and verbal processing, which weakens the conclusions that can be drawn from the findings. Nevertheless, there is mitigated support for the role of spatial working memory in mental rotation sex difference—for example, from a 2017 meta-analysis (Voyer et al., 2017). Hence, it seems sensible to investigate the relationship between working memory and mental rotation and the role of working memory in mental rotation sex differences more thoroughly.

In this context, a dual-task design, as used by Hyun and Luck (2007), is appealing because object and spatial working memory can be considered individually and are isolated via an articulatory suppression task. This approach further enables causal conclusions about the role of working memory in mental rotation. Nevertheless, some issues with the study by Hyun and Luck may be worth addressing. Their experiment included many more women than men (12/15 participants per experiment were women), precluding tests of sex effects and raising the possibility that their results reflect how women (but not men) maintain representations during mental rotation. Moreover, their study was potentially underpowered, given the small sample size, which leaves open the possibility of a Type 2 error as an alternative explanation for the results of Experiment 2. We conducted the present study as a replication and extension of the experiments conducted by Hyun and Luck to address these issues and clarify the role of working memory subsystems in mental rotation.

It should be noted that a previous attempt to replicate and extend the study of Hyun and Luck (2007) by (some of) the authors of this article failed to replicate the findings. However, as the experimental methods in the former experiment differed from those of the original study, no exact replication was achieved, and the results were never published. Precisely, next to minor deviations from the original experiment (which are unlikely to have had a significant impact on the data), the display of the mental rotation stimuli was 500 ms shorter than in the study by Hyun and Luck. The shorter stimulus presentation could have affected mental rotation response times and mental rotation accuracy as well as accuracy on the working memory tasks. Nevertheless, the same interference of visual working memory subsystems with the mental rotation process should have been tested in this experiment. Thus, despite the acknowledged differences, it is at least interesting that these unpublished results conflict with the theory of separate involvement of object and spatial working memory proposed by Hyun and Luck.

The current replication included a large number of men and women participants. In keeping with Hyun and Luck's (2007) findings, our working hypotheses regarding working memory interference were as follows: (1) Mental rotation should be slower and/or less accurate under dual-task conditions when the secondary task targets object working memory (Experiment 1). This effect should become more pronounced with increasing rotation angles. Object working-memory accuracy should be affected in the same way. (2) Potential task condition by angle of rotation interactions should be less pronounced (compared with Experiment 1) or even absent when the secondary task targets spatial working memory (Experiment 2). (3) In the joint data analyses, significant three-way interactions between task condition, angle of rotation, and experiment would be expected. Due to concerns about the statistical power to detect such three-way interactions at the achieved sample size, the analyses pertaining to this hypothesis are deemed exploratory.

Although our key focus is on a replication of the study by Hyun and Luck (2007), an extension to their work that investigates potential sex effects would be both sensible and interesting. As hinted before, a meta-analysis by Voyer et al. (2017) suggests that visuospatial working memory capacity is lower in women than men. Lower working memory capacity could make it harder to deal with increasing demands placed on these working memory systems. Hence, dual-task interference effects would be more pronounced in women than men. To address this hypothesis, we tested for three-way interactions between sex, task condition, and angular disparity. These analyses are deemed exploratory (see also the previous paragraph).

Moreover, it could be informative to look at potential sex effects per experiment and investigate whether they differ between experiments (i.e., test four-way interactions between sex, experiment, condition, and angular disparity). However, confirmatory analyses into such effects were not well powered in the context of this study. Therefore, we opted for an exploratory investigation of four-way interactions in this study.

## Method

### Participants

In reaction-time experiments, effect sizes are typically around *d* = 0.1 (Brysbaert & Stevens, 2018) due to the considerable variance of reaction times. Effects of such small magnitude are not readily detected, but this issue can (to a certain degree) be remedied through repeated measurements. To detect within-subjects effects of *d* = 0.1, Brysbaert and Stevens (2018) recommend at least 1,600 usable observations per condition to achieve adequate power (i.e., 1 – ß = .8). With an expected accuracy of 80% (on the mental-rotation task) and 16 trials per condition, this translates to 125 participants per experiment (i.e., object working memory vs. spatial working memory) or a total of 250 participants across both experiments. Concerning accuracy data, it appears reasonable to consider effect sizes typical in psychological experiments of practical relevance. According to Brysbaert (2019), such effects are in the realm of *d* = 0.3–0.4, and these values should thus be used as the minimal effect size of interest in power analyses. According to a power analysis using Superpower in R (Lakens & Caldwell, 2021), a total sample size of 126 participants would be needed to detect two-way, within-subjects, within-experiment interactions (Angle × Condition) at *d* = 0.4, given a correlation of *r* =.66 between repeated measurements^2^ and a power of 1 − ß = 0.8. Based on these considerations and attempting to account for possibly lower accuracy, the exclusion of outliers, and to aim for an overpowered design, we terminated data collection at 270 participants. Sixteen participants from Experiment 1 were excluded because their accuracy in at least one of the single-task conditions was below 60%. Data from two participants in Experiment 2 were missing entirely, either due to human error or technical problems. Additionally, 39 participants from Experiment 2 were excluded with accuracy rates below 60% in at least one of the single-task conditions. Thus, our final sample comprised 213 adult participants between 18 and 38 (mean age = 22.25 years, *SD* = 2.76). Of the 119 participants in Experiment 1, 57 were men and 62 were women. Of the 94 participants in Experiment 2, 52 were men, 41 were women, and one did not disclose their sex. Data for both experiments were collected concurrently, and participants were randomly assigned to either Experiment 1 or Experiment 2. The experiments took place in a laboratory room at the Department of Sports Science at the University of Regensburg. Participants were mainly recruited via an online newsletter. Individuals younger than 18 years, older than 40 years, diagnosed with mental illness, on medication known to affect cognitive performance, and/or those with impaired color vision were not eligible to participate in the study. Demographic data (age, sex, handedness) were registered. Participants chose to be compensated with course credit (only applicable for students at the University of Regensburg) or through participation in a lottery to win one of 20 shopping vouchers (worth 20€ each).

### Materials

**Fig. 1:**
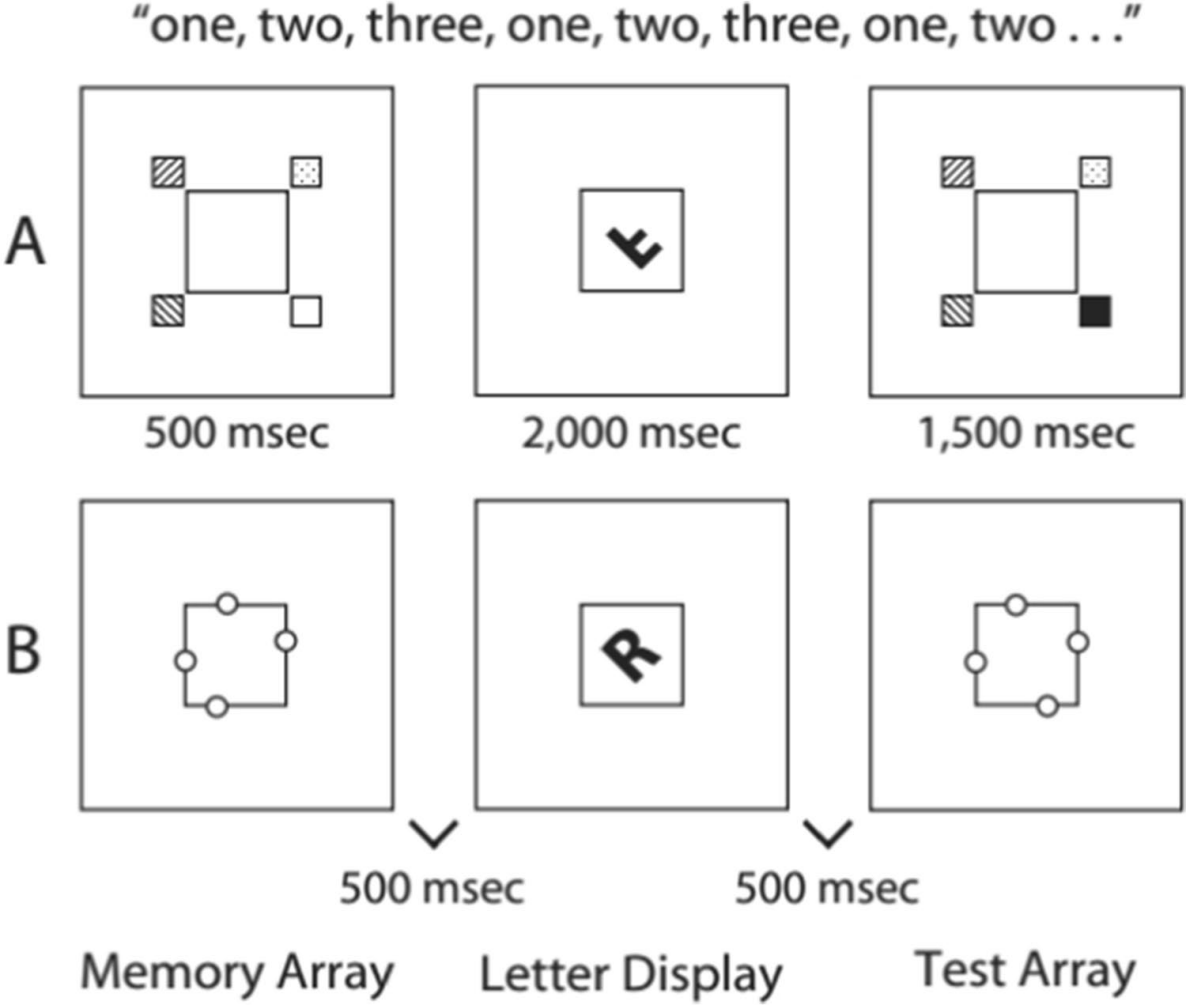
Example stimuli and procedure used in Experiments 1 and 2. (**A**) Stimuli and procedure used used in Experiment 1. Squares filled with patterns represent the colored items. (**B**) Stimuli and procedure used in Experiment 2. Dots were colored in white, and the placeholder box at the center was outlined in a light gray. The stimuli were presented on a gray background. The text at the top represents the concurrent articulatory suppression task that was used in both experiments. *Note.* Reprinted with permission from Nature/Springer/Palgrave, *Psychonomic Bulletin & Review*, Visual working memory as the substrate for mental rotation, by Hyun and Luck, 2007

The experiments were run in OpenSesame (Mathôt et al., 2012) with a gray background. A 15-in. Full HD (1,920 × 1,080) laptop screen was used at a viewing distance of approximately 70 cm. An external computer keyboard placed in front of the laptop was used to collect responses. Throughout the experiment, a light gray placeholder box (6.1° × 6.1°) was visible in the center of the computer display (see Fig. 1, reproduced from Hyun & Luck, 2007). Two different tasks (a mental-rotation task and a working-memory task) were combined to produce three different conditions: A mental-rotation-alone condition, a working-memory-alone condition, and a dual-task condition combining both tasks.

#### Mental-rotation task

In the mental-rotation task, black letters appeared in the middle of the placeholder box with a visual angle of approximately 2° wide × 2.5° high. The letters (F, J, G, P, R, Q, a, h, k, and t) were presented in random order at different angles of rotation (0°, 72°, or 144° in either direction in the picture plane from an upright position), either in canonical or mirror-reversed form and shown for 2,000 ms with a complete counterbalancing of letters and angles. Participants were instructed to make a speeded response (is the letter canonical or mirror reversed?) on a computer keyboard with the index or middle finger of their dominant hand.

#### Working memory tasks

Two distinct change-detection tasks assessed object and spatial working memory. The tasks consisted of a memory array and a test array, respectively. The test array was identical to the memory array in 50% of the trials and different in the remaining trials. During the presentation of the test array, participants were asked to indicate whether this array differed from the memory array. Both tasks were adopted from Hyun and Luck (2007) and are described in detail in the following sections.

##### Experiment 1: Object working memory task

The stimuli in the object working memory task were four colored squares (1.4° × 1.4°), each centered 2° diagonally at the corner of the central placeholder box. The seven possible colors for the squares were blue, green, black, red, violet, white, and yellow, with the color of each square selected at random from that set on each trial. Up to two squares could be the same color on a given trial. On change trials, the color of one randomly selected square was changed in the test array (after the mental rotation display) compared with the memory array (before the mental rotation display). In response to the test array, participants made an unspeeded response with the index or middle finger of their nondominant hand, indicating either change (1) or no change (2) compared with the memory array.

##### Experiment 2: Spatial working memory task

For the spatial working memory task, stimuli consisted of four white dots (0.16° × 0.16°), each on one side of the central placeholder box. The dots were placed randomly at a position ranging from 0° to 0.74° from the midpoint of each side of the box. On change trials, one dot in the test array was displaced by 0.98° from its original position (i.e., its position in the memory array). In response to the test array, participants made an unspeeded response with the index or middle finger of their nondominant hand, indicating either a change (1) or no change (2) compared with the memory array.

### Procedure

Participants completed three blocks (i.e., one mental-rotation-alone block, one working-memory-alone block, and one dual-task block) of experimental trials, each consisting of 48 trials. The block order was counterbalanced. In each trial, participants first saw the memory array for 500 ms followed by a single letter relevant to the mental-rotation task (i.e., the letter display) for 2,000 ms, followed by the test array for 1,500 ms that was either identical to or different from the initial memory array. A 500-ms delay preceded both the letter display and the test array, and there was a 1,500-ms intertrial interval. In the rotation-alone block, participants performed the letter rotation task and ignored the memory stimuli. In the memory-alone block, they performed the memory task but were instructed to ignore the mental rotation stimuli. In the dual-task block, they performed both tasks.

Additionally, following the approach used by Hyun and Luck (2007), participants performed an articulatory suppression task by continuously repeating out loud “one, two, three” (in German, “eins, zwei, drei”) throughout each trial of each condition. An experimenter was always present during task completion to ensure that participants were completing the task as requested.

### Data analysis

We analyzed data from each experiment in isolation (as did Hyun & Luck, 2007) as a first step. We further analyzed combined data from Experiments 1 and 2, although these analyses remained exploratory. Accuracy on the mental rotation and working memory tasks and mental rotation response time were used as dependent variables in separate mixed-model analyses. For the replication, block (single-task mental rotation, single-task working memory, dual task) and angular disparity (0°, 72°, 144°) were used as independent variables. Data for the rotation task was collapsed across canonical and mirror-reversed items. In all analyses, the variable block was treated as a factor. The single-task conditions were coded as −0.5 and the dual-task condition as 0.5. The angular disparity was coded numerically (centered around 72° and scaled so that −1 = 0°) and treated as a continuous variable. To extend the work of Hyun and Luck, we added sex (exploratory) as an independent variable. This variable was also treated as a factor. Since one participant did not disclose their sex, analyses including sex effects were conducted separately from the rest of our analyses. For the joint data analysis, experimental condition (Experiment 1 vs. Experiment 2) was additionally entered as an independent variable and treated as a factor. Experiment 1 was coded as 0.5 and Experiment 2 was coded as −0.5. All potential main and interaction effects were entered into our statistical models. However, our analyses focused on interaction effects that included the Block × Angular Disparity interaction, as these effects would imply process interference between working memory and mental-rotation tasks. To ensure ease of interpretation of main effects and lower-order interactions as part of higher-order interactions (this can be an issue in mixed-model approaches), all factors were sum coded (Levy, 2014). To ensure that the planned analyses were feasible, we simulated data and “analyzed” them using generalized linear mixed models (GLMMs) for binomial, gamma, and inverse Gaussian distributions prior to data collection. We ran simulations following two distinct approaches (a detailed description of the simulations, including the simulated parameters and parameter estimates from the respective GLMMs, can be found in Supplement 1).

Following recommendations by Lo and Andrews (2015), we analyzed reaction time data using generalized linear mixed models (GLMMs). There are several advantages of using linear mixed models compared with traditional analyses of variance.^3^ Moreover, GLMMs allow for the analysis of reaction-time data without the need for transformation (e.g., inverse transformation), thereby precluding common issues linked to such transformation in the context of reaction times (see Lo & Andrews, 2015). In line with their recommendations, we compared a Gaussian, an inverse Gaussian, and a gamma distribution on the most complex (converging) models. We chose the best-fitting distribution (in terms of deviance) for further analysis. If, for a given analysis, the random intercepts-only model did not converge, we discarded that distribution for the respective analysis. Accuracy data were analyzed using GLMMs based on the binomial distribution as recommended by Dixon (2008). The random factor participant/subject was included in all models. Additionally, mental rotation stimulus was included as a random factor in models concerning mental rotation performance to “(1) estimate the extent to which mean responses vary across units of the random factor(s); (2) allow inferences about whether fixed effects generalize beyond the units sampled in the random factor(s); (3) remove variability in responses that are associated with the random factor(s) rather than the conditions of experimental interest (i.e., reduce Type I error rate)” (Lo & Andrews, 2015, p. 5).

Following the approach Hyun and Luck (2007) used, response times faster than 100 ms or slower than 3,000 ms were excluded from all analyses, and only correct mental rotation responses were considered in response-time analyses. However, no more trimming was applied, following the recommendations of Baayen and Milin (2010). In addition, participants with an accuracy of 60% or lower in the single-task conditions were removed to ensure that all included participants understood what was expected of them.

For all effects of interest, we report both the unstandardized effect sizes (i.e., absolute mean difference) and confidence intervals calculated using parametric bootstrapping with 1,000 simulations, as recommended by Baguley (2009) and Pek and Flora (2018). Unfortunately, there is no agreement regarding the way to compute standardized effect sizes (e.g., Cohen’s *d*) in linear mixed models (Feingold, 2009; Hedges, 2007; Rights & Sterba, 2019).

Model building was based on research by Barr et al. (2013), Bates et al. (2015), and Matuschek et al. (2017). Guided by their recommendations, we started with models including all relevant fixed effects, random intercepts, and slopes for every appropriate fixed effect. We then reduced the random effects structure using a backward selection approach. Using likelihood ratio tests (LRTs) with α = .2 (this alpha level places a lower penalty on model complexity than the Akaike information criterion and is a sensible criterion; Matuschek et al., 2017), we determined whether random correlations or random slopes could be excluded from a given model without a significant decrease in model fit (i.e., slopes for which *p* > .2 will be excluded). We started with the random slopes of the highest order effects and excluded those effects yielding the least significant decrease in model fit one by one until further exclusion of any random slope yielded a significantly worse model. We conducted significance tests for all fixed effects based on the resulting models. Specifically, we employed LRTs comparing the final models to models excluding the fixed effects in question.

#### Deviations from the registered plan

Since bootstrapping confidence intervals could not be calculated for inverse Gaussian and gamma distributions, we report Wald confidence intervals where these distributions were used. Our registered plan was ambiguous regarding the random factor “stimulus.” We did not specify whether this referred to the mental rotation stimulus, the working memory stimulus, or both. In our analyses, only the letter participants saw for the mental-rotation task was included as the random factor stimulus. Moreover, this random factor was only included in analyses concerning mental rotation, contrary to our registered plan, which specified its inclusion in all analyses. To facilitate quicker data collection, the number of shopping vouchers that we gave away was increased from five to 20.

## Results

**Fig. 2:**
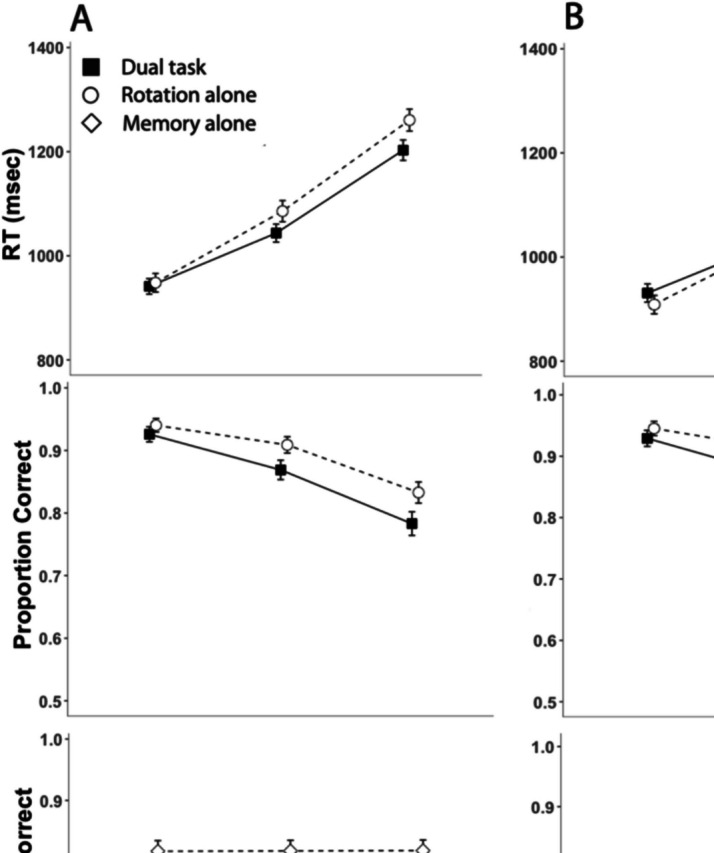
Outcomes as a function of rotation angle and task condition. *Note.* Results from Experiment 1 (**A**) and Experiment 2 (**B**). Top and middle rows show mental-rotation response-time data and proportion correct on the mental-rotation task, respectively. The bottom row indicates proportion correct on the working-memory tasks

The results are shown in Fig. 2A (Experiment 1) and Fig. 2B (Experiment 2).

### Experiment 1: Object working memory

Table 1 contains the model coefficients and associated test statistics of the GLMM on response time in Experiment 1. The rotation rate was 549°/s, and there was a significant main effect of rotation angle. Response times increased with greater rotation angles. The interaction between condition and rotation angle was significant. The rotation rate was 497°/s in the single-task condition and 613°/s in the dual-task condition. Thus, participants rotated stimuli faster in the dual-task condition than in the single-task condition.

**Table 1:** GLMM for dependent-variable mental-rotation response time—Experiment 1

Predictor	Estimate	*SE*	95% CI		Test statistic	*p* value
Intercept	1,410.05	12.64	1,380.12	1,429.66		
Condition	−1.21	8.31	−17.50	15.09	χ^2^(1) = 0.16	.688
Rotation	131.08	2.94	125.32	136.83	χ^2^(1) = 1751.99	<.001
Cond. × Rot.	−27.31	5.53	−38.14	−16.47	χ^2^(1) = 19.94	<.001

*Note.* This model followed an inverse Gaussian distribution and included random intercepts by stimulus, and participant and random slopes for condition by participant without correlations between random effects. Bootstrapping intervals could not be obtained. Wald confidence intervals are reported. *Cond.* Condition, *Rot.* Rotation

Model coefficients and test statistics of the GLMM on mental rotation accuracy in Experiment 1 are shown in Table 2. There was a significant main effect of rotation. Mental rotation accuracy decreased with greater rotation angles. No other effect reached significance in this model.

The GLMM for working-memory accuracy in Experiment 1 is summarized in Table 3. There was a significant main effect of condition. Average working-memory accuracy was lower in the dual-task condition compared with the working-memory-alone condition. No other effect reached significance in this model.

Our exploratory analyses did not reveal any significant sex effects (main effects or interactions including sex) in Experiment 1 (all *p* values > .15).

**Table 2:** GLMM for dependent-variable mental-rotation accuracy—Experiment 1

Predictor	Estimate	*SE*	95% CI		Test statistic	*p* value
Intercept	2.511	0.217	2.10	2.94		
Condition	−0.194	0.107	−0.41	0.03	χ^2^(1) = 3.02	.082
Rotation	−0.743	0.058	−0.86	−0.64	χ^2^(1) = 110.60	<.001
Cond. × Rot.	− 0.095	0.085	−0.27	0.09	χ^2^(1) = 1.17	.279

*Note.* This model included random intercepts by stimulus and participant and random slopes for rotation angle and condition by participant with correlations between random effects. *Cond.* Condition, *Rot.* Rotation

**Table 3:** GLMM for dependent-variable working-memory accuracy—Experiment 1

Predictor	Estimate	*SE*	95% CI		Test statistic	*p* value
Intercept	1.229	0.055	1.12	1.35		
Condition	−0.718	0.067	−0.86	−0.58	χ^2^(1) = 80.36	<.001
Rotation	−0.054	0.032	−0.12	0.01	χ^2^(1) = 2.75	.097
Cond. × Rot.	− 0.089	0.058	−0.20	0.03	χ^2^(1) = 2.35	.125

*Note.* This model included random intercepts by participant and random slopes for rotation angle and condition by participant with correlations between random effects. *Cond.* Condition, *Rot.* Rotation

### Experiment 2: Spatial working memory

Table 4 contains the model coefficients and associated test statistics of the GLMM on response time in Experiment 2. The rotation rate was 530°/s, and there was a significant main effect of rotation. Participants took more time to respond when rotation angles were greater. The interaction between condition and rotation was significant. The rotation rate was 473°/s in the single-task condition and 603°/s in the dual-task condition. Participants rotated stimuli faster in the dual-task condition than in the single-task condition.

The GLMM for mental rotation accuracy in Experiment 2 is summarized in Table 5. In contrast to Experiment 1, there was a significant main effect of condition. Average mental rotation accuracy was lower in the dual-task condition compared to the mental rotation only condition. There was also a significant main effect of rotation angle. Higher angles of rotation were associated with lower accuracy. The interaction between condition and rotation angle was not significant. 

Model coefficients and test statistics of the GLMM on working-memory accuracy in Experiment 2 are shown in Table 6. There was a significant main effect of condition. Working memory accuracy was lower in the dual-task condition than in the working-memory-alone condition. No other effect reached significance in this model.

Our exploratory analyses did not reveal any significant sex effects in Experiment 2 (all *p* values > .05).

**Table 4:** GLMM for dependent-variable mental-rotation response time—Experiment 2

Predictor	Estimate	*SE*	95% CI		Test statistic	*p* value
Intercept	1,116.04	4.43	1,107.35	1124.72		
Condition	−4.93	3.38	−11.55	1.69	χ^2^(1) = 0.73	.392
Rotation	135.851	2.61	130.74	140.96	χ^2^(1) = 1440.54	<.001
Cond. × Rot.	−32.91	3.24	−39.26	−26.55	χ^2^(1) = 23.05	<.001

*Note.* This model followed a gamma distribution and included random intercepts by stimulus and participant. Confidence intervals based on bootstrapping of linear mixed model. Bootstrapping intervals could not be obtained. Wald confidence intervals are reported. *Cond.* Condition, *Rot.* Rotation

**Table 5:** GLMM for dependent-variable mental-rotation accuracy—Experiment 2

Predictor	Estimate	*SE*	95% CI		Test statistic	*p* value
Intercept	2.459	0.224	2.00	2.90		
Condition	−0.408	0.100	−0.62	−0.22	χ^2^(1) = 15.28	<.001
Rotation	−0.690	0.063	−0.81	−0.57	χ^2^(1) = 79.29	<.001
Cond. × Rot.	− 0.028	0.091	−0.23	0.16	χ^2^(1) = 0.09	.764

*Note.* This model included random intercepts by stimulus and participant and random slopes for rotation angle and condition by participant without correlations between random effects. *Cond.* Condition, *Rot.* Rotation

**Table 6:** GLMM for dependent-variable working-memory accuracy—Experiment 2

Predictor	Estimate	*SE*	95% CI		Test statistic	*p* value
Intercept	0.646	0.040	0.57	0.73		
Condition	−0.466	0.073	−0.60	−0.33	χ^2^(1) = 33.22	<.001
Rotation	−0.022	0.028	−0.08	0.03	χ^2^(1) = 0.61	.435
Cond. × Rot.	− 0.060	0.056	−0.17	0.05	χ^2^(1) = 1.14	.285

*Note.* This model included random intercepts by participant and random slopes for condition by participant with correlations between random effects. *Cond.* Condition, *Rot.* Rotation

### Joint data analysis

Table 7 shows both experiments' model coefficients and test statistics for the LMM on response time data. Our analysis confirmed a significant main effect of rotation angle and a significant interaction between task condition and rotation across experiments. No effects involving experiment were found.

Table 8 summarizes the GLMM on mental-rotation accuracy across experiments.

There were significant main effects of task condition and rotation angle. Mental-rotation accuracy was lower in the dual-task condition across experiments and decreased with increasing rotation angles. No effects involving experiment were found. 

Model coefficients and associated test statistics for the GLMM on working-memory accuracy across experiments are shown in Table 9. There was a significant effect of task condition on working-memory accuracy. Working-memory accuracy was lower in the dual-task condition in both experiments. There was also a significant main effect of experiment. Working-memory accuracy was higher in Experiment 1 than in Experiment 2. The joint analysis also showed a significant interaction effect of task condition and experiment. The difference between accuracy in the working-memory-only condition and the dual-task condition was larger in Experiment 1 than Experiment 2. No other effects were found.

Exploratory analyses of data from both experiments revealed no significant main effects of sex or any significant interaction effects including sex on any dependent variables (all *p* values > .10).

**Table 7:** LMM for dependent-variable mental-rotation response time

Predictor	Estimate	*SE*	95% CI			Test statistic	*p* value
Intercept	1,087.00	29.57	1,028.50	1,146.62			
Condition	−4.76	4.37	−13.10		4.28	χ^2^(1) = 1.19	.276
Rotation	144.52	2.67	139.07		149.69	χ^2^(1) = 2699.76	<.001
Experiment	44.96	36.40	−27.10		114.48	χ^2^(1) = 1.52	.218
Cond. × Rot.	− 33.88	5.34	−44.26		−24.38	χ^2^(1) = 40.22	<.001
Cond. × Exp.	−1.14	8.75	−18.28		15.57	χ^2^(1) = 0.02	.897
Rot. × Exp.	1.24	5.34	−8.88		11.70	χ^2^(1) = 0.05	.817
Cond × Rot. × Exp.	10.67	10.68	−10.86		31.44	χ^2^(1) = 1.00	.318

*Note.* This model included random intercepts by stimulus and participant. *Cond.* Condition, *Rot.* Rotation, *Exp.* Experiment

**Table 8:** GLMM for dependent-variable mental-rotation accuracy

Predictor	Estimate	*SE*	95% CI		Test statistic	*p* value
Intercept	2.456	0.212	2.08	2.85		
Condition	−0.429	0.101	−0.63	−0.22	χ^2^(1) = 12.82	<.001
Rotation	−0.692	0.030	−0.76	−0.63	χ^2^(1) = 543.2	<.001
Experiment	0.021	0.112	−0.20	0.24	χ^2^(1) = 0.03	.852
Cond. × Rot.	− 0.022	0.061	−0.15	0.10	χ^2^(1) = 0.12	.724
Cond. × Exp.	0.116	0.145	−0.16	0.42	χ^2^(1) = 0.62	.430
Rot. × Exp.	−0.018	0.060	−0.15	0.11	χ^2^(1) = 0.09	.764
Cond. × Rot. × Exp.	−0.056	0.121	−0.31	0.18	χ^2^(1) = 0.21	.648

*Note.* This model included random intercepts by stimulus and participant and random slopes for condition by stimulus and participant without correlations between random effects. *Cond.* Condition, *Rot.* Rotation, *Exp.* Experiment

**Table 9:** GLMM for dependent-variable working-memory accuracy

Predictor	Estimate	*SE*	95% CI		Test statistic	*p* value
Intercept	0.934	0.035	0.87	1.00		
Condition	−0.583	0.049	−0.68	−0.47	χ^2^(1) = 106.69	<.001
Rotation	−0.040	0.022	−0.08	>0	χ^2^(1) = 3.38	.066
Experiment	0.556	0.070	0.43	0.69	χ^2^(1) = 56.32	<.001
Cond. × Rot.	−0.073	0.040	−0.15	0.01	χ^2^(1) = 3.26	.071
Cond. × Exp.	−0.201	0.098	−0.38	−0.01	χ^2^(1) = 4.16	.041
Rot. × Exp.	−0.031	0.043	−0.12	0.06	χ^2^(1) = 0.51	.475
Cond × Rot. × Exp.	−0.037	0.080	−0.19	0.12	χ^2^(1) = 0.22	.642

*Note.* This model included random intercepts by participant and random slopes for rotation angle and condition by participant with correlations between random effects. *Cond.* Condition, *Rot.* Rotation, *Exp.* Experiment

## Discussion

The results of Hyun and Luck (2007) suggested that object working memory is responsible for holding object representations during mental rotation, whereas spatial working memory is not. However, the findings of our replication study do not support this notion. Where we observed interference, there were no differences in the extent of interference between the group that memorized object colors and the group that memorized spatial locations. Moreover, visuospatial working memory does not seem to be relevant to the rotation process in the mental rotation of letters, as any dual-task interference found was independent of the degree of rotation. This pattern of results suggests that neither visuospatial working memory, in general, nor object working memory, specifically, are relevant to the mental rotation of letters in the way Hyun and Luck assumed.

According to capacity-sharing models (and similar bottleneck models), dual-task interference results from two tasks or processes involved in these tasks, relying on the same mental resources or mechanisms (Pashler, 1994). In the context of this study, this would mean that mental rotation involves cognitive resources that are also relevant to working-memory tasks. Here, we assume that visuospatial working memory itself is one of these shared resources.^4^ The notion of visuospatial working memory involvement fits well with the discussed correlational findings (Kaufman, 2007; Lehmann et al., 2014), and neuroimaging studies that implicate frontal brain areas associated with visuospatial working memory and attention (e.g., Awh & Jonides, 2001; Cohen et al., 1996; Mayer et al., 2007) in mental rotation (Gogos et al., 2010). As Hyun and Luck (2007) note, neuroimaging studies further suggest the involvement of the dorsal “where” pathway in the rotation process (see, e.g., Gogos et al., 2010). On the other hand, the ventral “what” stream is often implicated in object processing and recognition (Koshino et al., 2005). As Koshino et al. (2005) point out, the tasks are not carried out by these systems in isolation. Instead, areas of the dorsal and ventral streams play a part in multiple processes of mental rotation, albeit with distinct relative specializations. Interestingly, Koshino et al. observed different brain activation patterns depending on stimulus complexity. With simple stimuli, increased rotation was linked to increased parietal activation only. However, the rotation of more complex stimuli elicited higher activation in regions across the cortex, including regions linked to visuospatial working memory. Hence, visuospatial working memory may serve a relatively limited role in the mental rotation of simple stimuli (such as letters) but be crucial in cases of more complex stimuli. This explanation aligns with our results, indicating a general, rotation-independent involvement of visuospatial working memory in the mental rotation of letters. Here, visuospatial working memory appears relevant to stages other than the rotation process. That is, working memory may be employed during the perceptual and/or the decision-processing stages. As later perceptual stages of mental rotation involve keeping a stimulus in mind and manipulating it, the involvement of working memory appears likely. Following a similar line of thought, Podzebenko et al. (2002) argued that visuospatial working memory may be relevant for comparing a stimulus and a known target. The findings of Koshino et al. (2005) highlight that our conclusions may not apply to mental rotation in general but rather to the mental rotation of simple stimuli. Testing the involvement of visuospatial working memory in the mental rotation of complex stimuli (e.g., complex polygons or 3D block figures) using a dual-task approach would be informative.

Our exploratory analyses did not reveal any sex differences. Further investigations into potential factors involved in mental rotation sex differences are warranted. However, it may be most sensible to focus on tests where sex differences are frequently observed (e.g., the Mental Rotations Test; Vandenberg & Kuse, 1978). As Jost and Jansen (2024) point out, sex differences (on psychometric tests) are typically smaller when simpler stimuli are used, which may also play a role in the context of this study. Examining differences between tests that produce sex differences and those that do not may provide guidance in identifying relevant factors (Jost & Jansen, 2024).

Two unexpected observations should be addressed. Firstly, it is surprising that rotation was slower in the single-task condition than in the dual-task condition across experiments. Unfortunately, standardized effect sizes are not available for context (see also the Method section). It should also be noted that the accuracy on both working-memory tasks was considerably lower than in the study by Hyun and Luck (2007). This discrepancy may be rooted in differences between the samples. However, it may also have reasons that could explain our failure to replicate the dual-task interference observed by Hyun and Luck. For example, it is possible that participants in our study did not dedicate much effort to solving the working-memory tasks and, as a result, did not use their full working-memory capacity for these tasks. If that were the case, they could have used the spare working-memory capacity to solve the mental-rotation task in the dual-task condition. Nevertheless, this explanation does not align well with the observed dual-task interference in working-memory performance.

In conclusion, we could not replicate the findings of Hyun and Luck (2007). Our results suggest that visual and spatial working memory may be involved in processes other than rotation in simple mental-rotation tasks. This is in line with evidence from neuroimaging studies. The role of visuospatial working memory in mental-rotation tasks involving complex stimuli should be tested experimentally, for example, using a dual-task design.

## Supplementary Information

Below is the link to the electronic supplementary material.

Supplementary file1 (PDF 151 KB)

Supplementary file2 (R 4 KB)

Supplementary file3 (R 4 KB)

Supplementary file4 (R 7 KB)

## Data Availability

The authors made raw anonymized data available via the Open Science Framework (https://osf.io/7x4rd/files/osfstorage).

## Footnotes

1 Although this work focuses on the role of working memory systems, it should be noted that short-term memory and working memory are hard to disentangle. Prominent models of working memory (e.g., Baddeley, 2000; Cowan, 2008) typically include short-term memory (i.e., maintenance of information without manipulation) as a component of working memory (i.e., maintenance and manipulation of information).

2 This is likely a relatively low estimate based on the comparison of accuracy in different tests of Voyer et al. (2006). Actual correlations should be higher, especially for reaction time (Brysbaert, 2019).

3 For instance, trial-level data are used as the unit of analysis, which prevents loss of information from averaging across participants and items. This approach also allows the simultaneous analysis of by-participants and by-item variance, precluding the need for separate by-participants and by-items analyses and resulting in greater statistical power. In addition, unlike an ANOVA, LMM is unaffected by an imbalance in the design. The consideration of a large data set reflecting the number of participants and the number of trials in a task also minimizes the influence of statistical outliers. For a more detailed discussion of these advantages, and others omitted here, interested readers should consult Baayen et al. (2008) as well as Boisgontier and Cheval (2016).

4 We note that the possibility of other shared resources or cross-talk (see Pashler, 1994) underlying the interference cannot be ruled out.

## Code availability

The analysis scripts are publicly available via the Open Science Framework (https://osf.io/7x4rd/files/osfstorage).
