# Supplementary material for: Visual working memory as the substrate for mental rotation: A replication
Source: Psychon Bull Rev. 2024 Dec 2;32(3):1204–16. doi: 10.3758/s13423-024-02602-4 (PMC12092522; doi:10.3758/s13423-024-02602-4)
Supplement: Supplementary file 1 — Supplementary file1 (PDF 151 KB) [file 13423_2024_2602_MOESM1_ESM.pdf]

Supplement 1: Description of Simulations and Parameter Estimates

Two simulations were conducted to demonstrate the appropriateness of the planned analyses. As described in more detail below, we used two approaches: 1) simulation of datasets based on arbitrarily chosen effects, and extraction of the associated parameters and 2) simulation of datasets based on Hyun and Luck (2007) and extraction of the parameters. In both simulation approaches, we ran 100 simulations (in approach 2 per experiment) for each of the selected non-normal distributions (Binomial, inverse Gaussian, and Gamma distribution). We begin with a detailed description of our original approach to the simulation (using arbitrary effects): We used a sample size of N=250. As between-subjects factors, we entered sex with two levels and condition (experiment) with two levels with a quarter of the simulated participants in each combination of these factors. Additionally, two within-subjects variables were generated. Angle as a numerical variable with three levels and block (single task v dual-task) as a factor with two levels. For each combination of within-factors, 20 observations were generated. We arbitrarily chose five fixed effects to deviate from zero. For the Gamma and inverse Gaussian distribution, the intercept was set to **1000**, the main effect of sex was set to **40**, the main effect of angle was set to **40**, the main effect of block was set to **60**, and the effect for the interaction of sex and angle was set to **20**. For the logarithmic odds underlying the Binomial distribution, the effects were set to (in the same order) **3, 1, 2, 3**, and **6**.

In each simulation, random data were then generated based on the specified mean effects. First, for each participant, a random intercept was generated from a standard normal distribution. For the Gamma and inverse Gaussian distribution, the random intercept was multiplied by 50 and was added to the effect of the respective condition to calculate the base value for each measurement. For the Gamma distribution, the final distribution was then calculated by using the base value of each measurement divided by 100 as the shape parameter and 0.01 as the rate parameter. For the inverse Gaussian distribution, the mean was set to the base value and the shape parameter was set to 10000. Consequently, both distributions had the base value as expected value and a variance of ~100,000 (at the average base value of 1000, the variance would be exactly 100,000). Based on the work of Lo and Andrews (2015), these values seem to be in the ballpark for reaction time measurements and despite the differences between mental rotation and word recognition we expect these variances to be in the order of realistic values. For the binomial distribution, the logarithmic odds were transformed to a probability based on which answers were randomly generated.

For each randomly generated data set, we then ran a GLMM analysis using the assigned distribution containing all main effects and interactions (up to the four-way interaction) and random intercepts by participants. The average values obtained through this procedure indicate that the fixed effects were estimated accurately. Table 1 contains mean estimates of all effects alongside standard errors. Overall, the results indicate that the Models were implemented correctly and are suitable for the planned analyses. The results are replicable using the seed function and the function can be adapted to simulate varying effect sizes.

We also decided to run simulations based on the data from HL2007. We used a very similar approach although only angle and block were introduced as centered variables in these

Table 1. True Values and Parameter Estimated (Standard Errors) of Arbitrary Fixed Effects

**Effect**	**Gamma Distribution**			**Inverse Gaussian Distribution**			**Binomial Distribution**		
	True Value	Estimate	(SE)	True Value	Estimate	(SE)	True Value	Estimate	(SE)
Intercept	1000.00	999.38	(3.71)	1000.00	997.11	(3.92)	3.000	2.999	(0.08)
Sex	40.00	41.02	(7.89)	40.00	39.50	(7.67)	1.000	1.001	(0.17)
Angle	40.00	39.93	(4.07)	40.00	40.26	(4.47)	2.000	2.003	(0.10)
Block	60.00	59.77	(3.91)	60.00	60.44	(3.50)	3.000	3.013	(0.10)
Exp	0.00	0.63	(7.04)	0.00	0.90	(7.88)	0.000	0.033	(0.17)
Sex*Angle	20.00	19.05	(8.13)	20.00	17.68	(7.98)	6.000	6.012	(0.21)
Sex*Block	0.00	0.23	(6.21)	0.00	3.60	(7.96)	0.000	0.001	(0.21)
Angle*Block	0.00	0.50	(8.12)	0.00	-3.63	(10.16)	0.000	0.014	(0.21)
Sex*Exp	0.00	-0.01	(15.10)	0.00	-2.09	(13.70)	0.000	0.012	(0.34)
Angle*Exp	0.00	-0.80	(9.70)	0.00	-1.00	(9.36)	0.000	0.003	(0.22)
Block*Exp	0.00	-0.79	(7.17)	0.00	0.23	(6.95)	0.000	-0.001	(0.29)
Sex*Angle*Block	0.00	-2.13	(16.44)	0.00	-3.80	(16.68)	0.000	0.032	(0.44)
Sex*Angle*Exp	0.00	-3.48	(19.21)	0.00	0.74	(18.76)	0.000	0.015	(0.44)
Sex*Block*Exp	0.00	3.40	(16.15)	0.00	-7.50	(13.94)	0.000	-0.021	(0.39)
Angle*Block*Exp	0.00	-2.56	(19.42)	0.00	8.82	(18.61)	0.000	-0.037	(0.45)
Sex*Angle*Block*Exp	0.00	1.49	(32.37)	0.00	13.62	(38.54)	0.000	0.000	(0.82)

simulations. Additionally, for these experiments we reduced the number of simulated participants to 150 and the number of observations (per participant, per angle, per block) was lowered to 16. Per experiment we then simulated mental rotation reaction time and mental rotation accuracy data. Akin to what was described above we specified what effects were to deviate from zero. For the object working memory experiment we set the intercept for reaction time data to **990**, the effect of angle to **220**, and the effect of block to **110**, the interaction effect of block and angle was set to **0**. The logarithmic odds were set to **3.3** (intercept), **-1** (angle), **0** (block), and **-1.5** (angle*block interaction). Based on these parameters, a pattern of data that resemble the data from HL2007 were created. For the spatial working memory experiment, we set the intercept for reaction time data to **1030**, the effect of angle to **300**, the effect of block to **-20**, and the interaction effect to **0**. The logarithmic odds were set to **3.6** (intercept), **-1** (angle), and **0** (block and angle*block interaction). To recreate the pattern from HL2007 (i.e.,

the steeper drop of accuracy with greater angular disparity), the levels of angle were coded as 0.00, 0.35, and 1.75 for the simulation of accuracy data in the spatial working memory experiment.

Again, we then ran a GLMM analysis using the assigned distribution on every randomly generated dataset. Table 2 contains mean estimates of all effects and the associated standard errors. The results indicate that the models were implemented correctly. R code for all simulations is attached to this submission. The code for the simulation using arbitrary effects can be found in "Simulation_Arbitrary.R", the code for the simulation based on the OWM experiment in Hyun and Luck (2007) can be found in "Simulation_OWM_HL.R", and the code for the simulation based on the SWM experiment in Hyun and Luck (2007) can be found in "Simulation_SWM_HL.R".

Table 2. True Values and Parameter Estimated (Standard Errors) of Fixed Effects Based on HL2007

**Experiment**	**Effect**	**Gamma Distribution**			**Inverse Gaussian Distribution**			**Binomial Distribution**		
		True Value	Estimate	(SE)	True Value	Estimate	(SE)	True Value	Estimate	(SE)
OWM	Intercept	990.00	992.25	(4.99)	990.00	995.01	(4.21)	3.300	3.298	(0.111)
	Angle	220.00	220.02	(6.13)	220.00	219.81	(2.92)	-1.000	-0.997	(0.135)
	Block	110.00	109.58	(4.14)	110.00	110.25	(1.93)	0.000	0.009	(0.090)
	Angle*Block	0.00	-0.40	(8.64)	0.00	0.23	(4.56)	-1.500	-1.500	(0.213)
SWM	Intercept	1030.00	1031.42	(4.39)	1030.00	1034.80	(4.09)	3.600	3.592	(0.111)
	Angle	300.00	301.13	(6.27)	300.00	300.50	(3.29)	-1.000	-0.994	(0.056)
	Block	-20.00	-20.28	(4.23)	-20.00	-20.22	(1.90)	0.000	-0.010	(0.109)
	Angle*Block	0.00	-0.94	(8.85)	0.00	-062	(4.64)	0.000	0.002	(0.084)
